# Exploration of common immune mechanisms and hub genes in latent and active tuberculosis infection

**DOI:** 10.3389/fcimb.2026.1798990

**Published:** 2026-05-20

**Authors:** Xingzhen Yang, Ming Chang, Fengzhen Liu

**Affiliations:** Department of Respiratory Medicine, Taian Cancer Hospital, Tai’an, China

**Keywords:** active tuberculosis infection, immune, latent tuberculosis infection, mycobacterium tuberculosis, nomogram

## Abstract

**Background:**

Tuberculosis continues to pose a severe global public health challenge. This study aims to explore the shared hub genes of latent tuberculosis infection (LTBI) and active tuberculosis (ATB).

**Methods:**

The common differentially expressed genes were obtained for LTBI and ATB, followed by PPI network construction for common genes and hub genes identification by LASSO regression analysis. The nomogram based on hub genes was established, and its predictive performance was evaluated by ROC curve analysis and decision curve analysis. Gene set variation analysis (GSVA) and immune characteristics evaluation were performed as well.

**Results:**

In total, 180 common genes were shared by LTBI and ATB. Based on PPI network and LASSO regression analysis, four hub genes were obtained, including C1QB, MSR1, OLIG3 and TGFB1I1. The nomogram risk prediction models for LTBI and ATB showed significant clinical net benefits across a broad range of threshold probabilities, indicating potential clinical application value. GSVA revealed a highly complex, multi-pathway coordinated immune activation pattern shared by LTBI and ATB. Gamma delta T cell and Type 17 T helper cell may be important participants in the tuberculosis immune response.

**Conclusion:**

This study identified hub genes shared by LTBI and ATB, laying a solid foundation for future molecular mechanism research and offering a novel perspective for the diagnosis and treatment of tuberculosis.

## 1 Introduction

Tuberculosis, caused by the human pathogen *Mycobacterium tuberculosis* (*M. tb*), remains one of the leading causes of mortality attributed to a single infectious agent (Möller et al., 2018). *M. tb* predominantly infects humans, with transmission occurring through aerosolized droplets (Churchyard et al., 2017). According to estimations from the World Health Organization, an estimated 10.7 million individuals worldwide fell ill with tuberculosis and 1.23 million died from the disease in 2024 (https://www.who.int/teams/global-programme-on-tuberculosis-and-lung-health/tb-reports/global-tuberculosis-report-2025). While pulmonary involvement is most common, tuberculosis is a multisystemic disease with highly variable clinical manifestations (Swinkels et al., 2025). The emergence of multiple drug-resistant tuberculosis and rising incidence of co-infection with human immunodeficiency virus (HIV) and tuberculosis have posed formidable challenges to tuberculosis control, particularly in low-income countries (Reid et al., 2023).

As a highly adapted intracellular pathogen, *M. tb* primarily targets macrophages and has evolved elaborate strategies to evade host immune surveillance and establish long-term persistence (Awuh and Flo, 2017). Infection can either be contained in the latent state, termed latent tuberculosis infection (LTBI), or progress to active tuberculosis (ATB) (Simmons et al., 2018). Approximately 5-10% of individuals infected with *M. tb* develop active disease within the first 2–5 years after infection (Carranza et al., 2020). LTBI is asymptomatic and may persist for decades before the onset of ATB (Simmons et al., 2018). Notably, LTBI and ATB represent distinct stages of the same infectious process induced by the identical pathogen, sharing certain underlying biological mechanisms (Drain et al., 2018; Gong and Wu, 2021).

In recent years, the emergence and widespread application of high-throughput sequencing technology have rendered bioinformatics an indispensable tool in the assessment of disease onset and progression, as well as in the identification of diagnostic and prognostic biomarkers. In the present study, we identified differentially expressed genes (DEGs) in LTBI and ATB, as well as DEGs common to both LTBI and ATB, using gene expression datasets retrieved from the GEO database, aiming to systematically explore the key molecular markers associated with LTBI and ATB, alongside their shared pathogenic mechanisms.

## 2 Materials and methods

### 2.1 Data collection

Using “homo sapiens”, “latent tuberculosis infection”, or “active tuberculosis infection” as the keywords, two datasets were gathered from the GEO database after excluding cell line/animal level studies and single sample studies. GSE19491 was served as a training set, including whole blood samples from 36 healthy controls (CON), 69 LTBI, and 61 ATB. GSE19444 was served as a validation set, including whole blood samples from 12 healthy controls (CON), 21 LTBI, and 21 ATB. After obtaining the expression matrices of the two datasets, platform annotation files were used to map the probes to gene symbols and perform log transformation on the expression values. For multiple probes corresponding to the same gene, the maximum expression value was retained as the final expression level of that gene (Wu et al., 2020; Yang and Xu, 2021). In addition, to mitigate the influence of extreme outliers that may be amplified by the maximum value, the data is subsequently subjected to logarithmic transformation. The characteristics of the individuals involved in two datasets were displayed in **Supplementary Table 1**. For more detailed information about the datasets, please refer to the original literature (Berry et al., 2010).

### 2.2 Differential expression analysis and protein-protein interaction network construction

The R package “limma” was used to obtain the DEGs in CON vs LTBI and CON vs ATB with *p*_adj *<*0.05 & |log_2_FC| ≥1. The R package “clusterProfiler” was applied to conduct functional enrichment analysis for DEGs in LTBI and ATB, as well as genes common to both LTBI and ATB (*p* < 0.05). Benjamini and Hochberg procedure was applied to adjust for multiple hypothesis testing. The PPI network for common genes was constructed based on STRING database (v12.0; https://string-db.org/) with interaction score ≥0.4 and visualized by Cytoscape software (v3.8.2).

### 2.3 Identification of hub genes

Based on the candidate genes in the PPI network, the R package “glmnet” was used to conduct LASSO regression analysis for further screening the hub genes, and the parameter λ was adjusted for 10-fold cross-validation. The Pearson correlation analysis was performed among these hub genes to further explore the potential connections. This analysis aimed to reveal the roles and potential associations of the hub genes in the common pathological mechanisms of LTBI and ATB, providing new clues for a deeper understanding of the common pathogenesis of LTBI and ATB.

### 2.4 Construction of the nomogram

The nomogram was constructed based on hub genes using the R package “rms”. Each gene was assigned a specific score, and the risk of LTBI and ATB was predicted based on the cumulative score of all included genes. Receiver operating characteristic (ROC) curve analysis was performed, then the area under the curve (AUC) was calculated to determine the discriminatory power of the nomogram. The consistency between the expected probability and the actual results was assessed using the calibration curve. Decision curve analysis (DCA) was applied to evaluate the clinical utility of the nomogram.

### 2.5 Gene set variation analysis and evaluation of immune characteristics

GSVA was performed to evaluate the potential biological functional changes of different samples. The gene set of “h.all.v2025.1.Hs.symbols.gmt” was downloaded from MSigDB database (https://www.gsea-msigdb.org/gsea/msigdb/) for running GSVA. A significant change was defined by a threshold of |t value of the GSVA score| greater than 1 (Zhang et al., 2022, 2025). To further clarify the potential relationship between hub genes and functional pathways, we also explored the correlation between hub genes and pathways using Pearson correlation analysis. The ssGSEA (single-sample gene-set enrichment analysis) algorithm was applied to quantify the relative abundance of each immune cell infiltration in LTBI and ATB. The correlations between hub genes and differentially infiltrating immune cell subsets were explored using Pearson correlation analysis to clarify the potential interaction between key molecules and the immune microenvironment in the pathological mechanisms of LTBI and ATB.

### 2.6 Real time qPCR validation of hub genes

Blood samples from 9 patients with ATB, 10 patients with LTBI and 10 controls were subjected to perform RT-qPCR. Total RNA from blood samples were extracted with blood RNA extraction kit according to the manufacturer’s protocol. We obtained the written informed consent and the approval of the ethics committee of Taian Cancer Hospital (TASZYYLL 2025019). The RT-qPCR was performed in Gene-9660 Real-time PCR Detection System with SuperReal PreMix Plus (SYBR Green) (TIANGEN, Beijing). Relative gene expression was analyzed by 2^-ΔΔCT^ method. GAPDH and ACTB were used as endogenous controls.

## 3 Results

### 3.1 Identification of DEGs in LTBI and ATB

Differential expression analysis revealed 687 DEGs (610 up-regulated and 77 down-regulated genes) between controls and LTBI (**Supplementary Figure 1A**). The GO analysis results showed that these DEGs were significantly enriched in multiple immune-related biological processes, including cellular response to chemokine, positive regulation of natural killer cell-mediated cytotoxicity, and chemokine-mediated signaling pathway, and multiple terms related to neural signal transduction and synaptic plasticity, such as axonogenesis, regulation of presynaptic membrane potential, acetylcholine-gated channel complex, acetylcholine receptor activity, and postsynaptic neurotransmitter receptor activity (**Supplementary Figure 1B**, **Supplementary Table 2**). The KEGG pathway analysis further confirmed these findings, with significantly enriched pathways such as “hsa04725: Cholinergic synapse”, “hsa04728: Dopaminergic synapse”, “hsa04724: Glutamatergic synapse”, “hsa04650: Natural killer cell mediated cytotoxicity”, and “hsa04612: Antigen processing and presentation” (**Supplementary Figure 1C**, **Supplementary Table 2**). The above results collectively suggest that the maintenance of LTBI is associated with robust activation of immune defense pathways, while the concurrent enrichment of neural-related terms warrants further validation.

Differential expression analysis also revealed 722 DEGs (599 up-regulated and 123 down-regulated genes) between controls and ATB (**Supplementary Figure 2A**). GO analysis indicated that these DEGs were enriched in immune-related biological processes, including defense response to virus, regulation of innate immune response, immune response-activating signaling pathway, and interferon-mediated signaling pathway, as well as apoptosis-related biological processes, such as pyroptosis (**Supplementary Figure 2B**, **Supplementary Table 3**). The KEGG pathway analysis further confirmed these findings, with significantly enriched pathways such as “hsa04610: Complement and coagulation cascades”, “hsa04620: Toll-like receptor signaling pathway”, “hsa04625: C-type lectin receptor signaling pathway”, “hsa04650: Natural killer cell mediated cytotoxicity”, “hsa04062: Chemokine signaling pathway”, “hsa04659: Th17 cell differentiation”, and “hsa04217: Necroptosis” (**Supplementary Figure 2C**, **Supplementary Table 3**). These results collectively indicate that the pathogenesis of ATB involves complex disruptions in the immune regulatory network and activation of cell death processes, providing important clues for a deeper understanding of the immune pathological mechanism of ATB.

**Figure 1:**
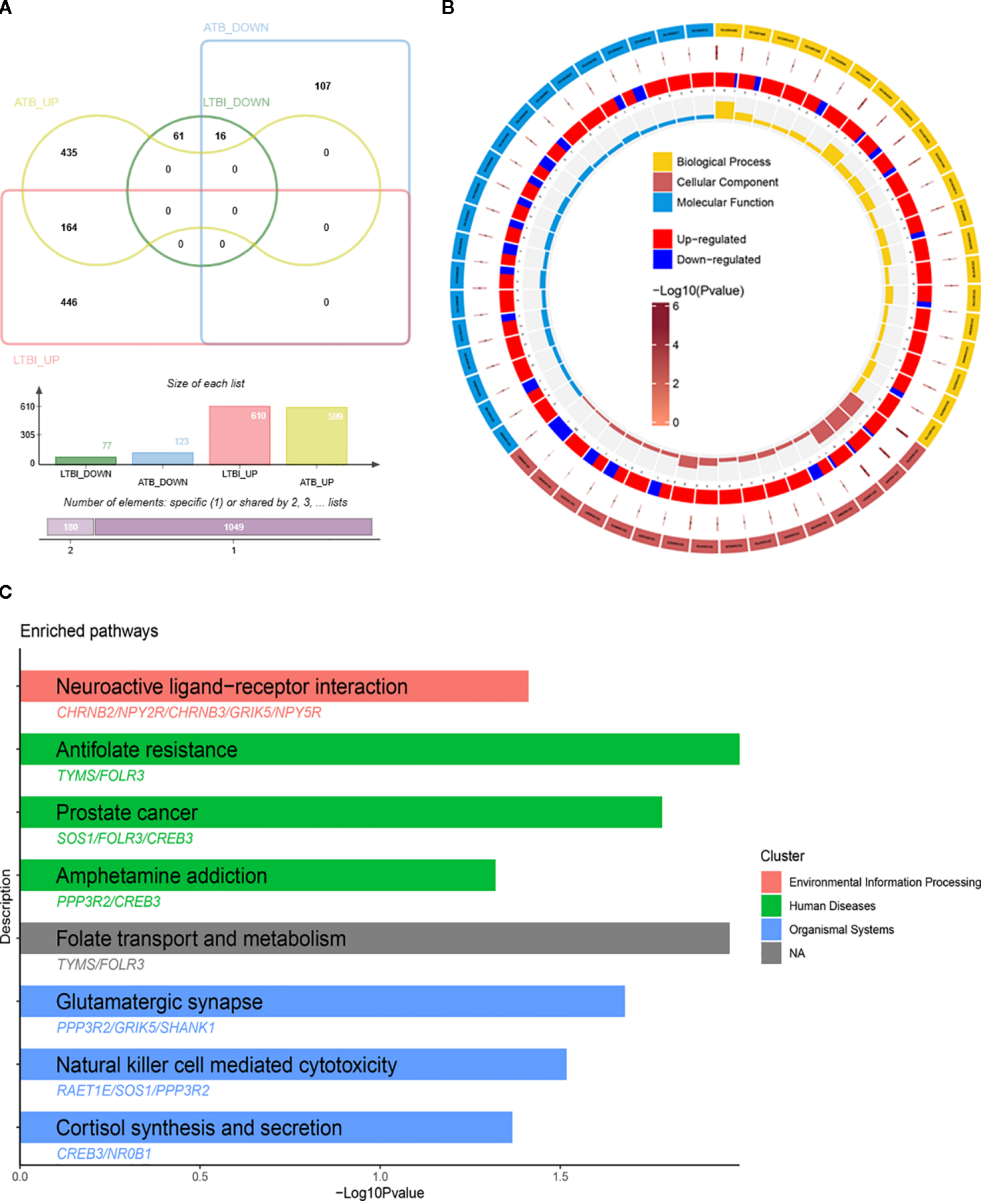
Identification and functional enrichment analysis of genes common to both LTBI and ATB. **(A)** Venn diagram showing the common genes. **(B)** GO analysis of common genes. **(C)** KEGG analysis of common genes.

In total, 180 common genes were obtained by taking the intersection of 610 up-regulated genes in LTBI and 599 up-regulated genes in ATB, as well as the intersection of 77 down-regulated genes in LTBI and 123 down-regulated genes in ATB, yielding 164 up-regulated and 16 down-regulated overlapping genes (**Figure 1A**). Similarly, GO analysis indicated that these common DEGs were enriched in neural signal transduction-related biological processes, such as central nervous system neuron differentiation, neurotransmitter receptor complex, postsynaptic neurotransmitter receptor activity, and acetylcholine receptor activity, and ion channel activity-related terms, such as sodium channel complex, potassium channel complex, cation channel complex, transmitter-gated monoatomic ion channel activity, transmitter-gated channel activity, extracellular ligand-gated monoatomic ion channel activity, and sodium channel activity (**Figure 1B**, **Supplementary Table 4**). The KEGG pathway analysis further indicated that these common genes were mainly involved in pathways related to neural signaling systems such as “hsa04724: Glutamatergic synapse” and “hsa04080: Neuroactive ligand-receptor interaction”, as well as pathways related to immunity and inflammatory responses such as “hsa04650: Natural killer cell mediated cytotoxicity” (**Figure 1C**, **Supplementary Table 4**). These results collectively revealed that the 180 common DEGs may play important roles in tuberculosis infection, laying a foundation for subsequent studies on the molecular mechanism of tuberculosis.

### 3.2 Identification of hub genes

**Figure 2:**
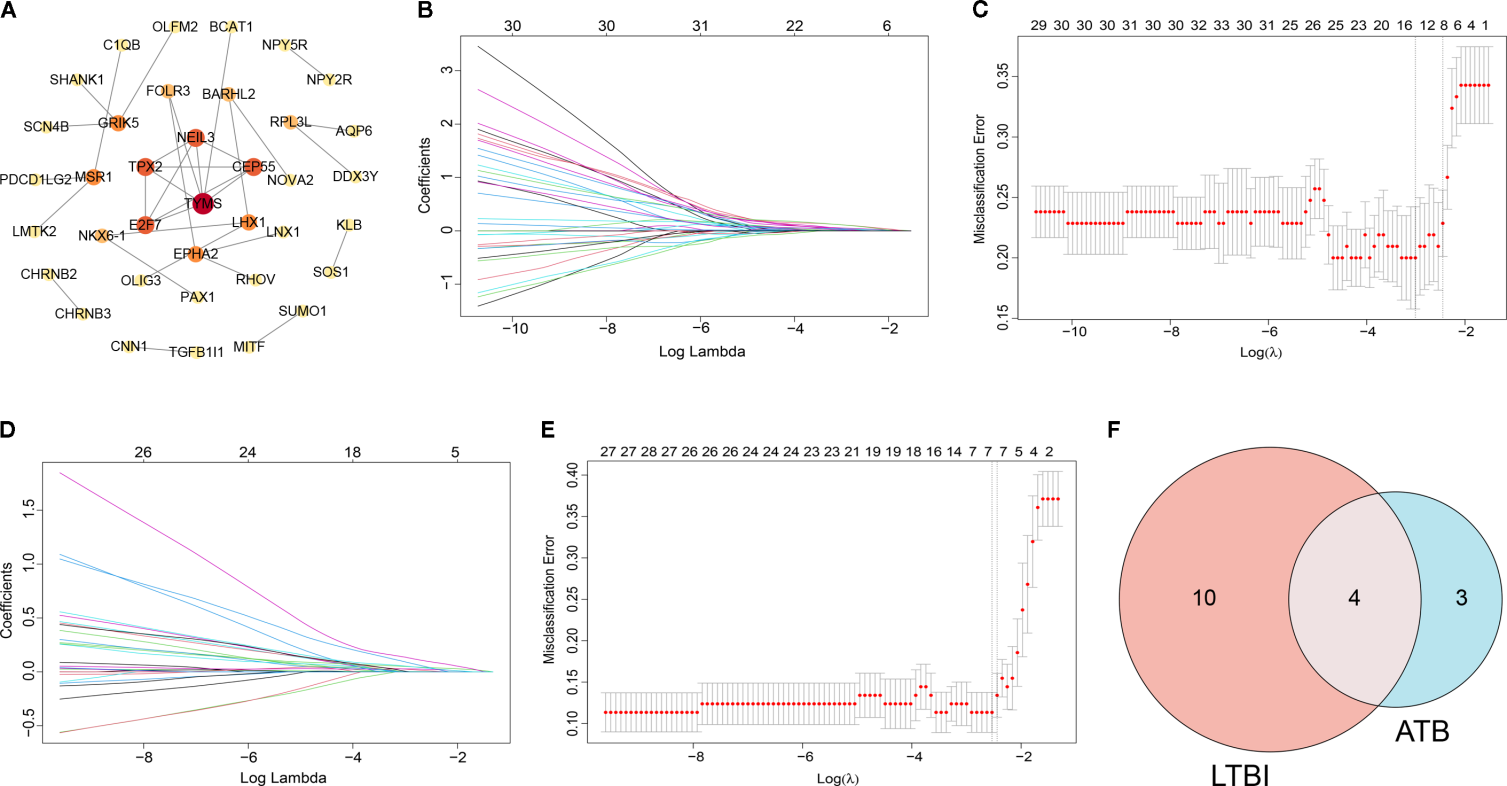
Identification of hub genes in LTBI and ATB. **(A)** protein-protein interaction network of common genes. **(B, C)** Identification of key genes in LTBI by LASSO analysis. **(D, E)** Identification of key genes in ATB by LASSO analysis. **(F)** Venn diagram represented the overlap between key genes in LTBI and ATB.

**Figure 3:**
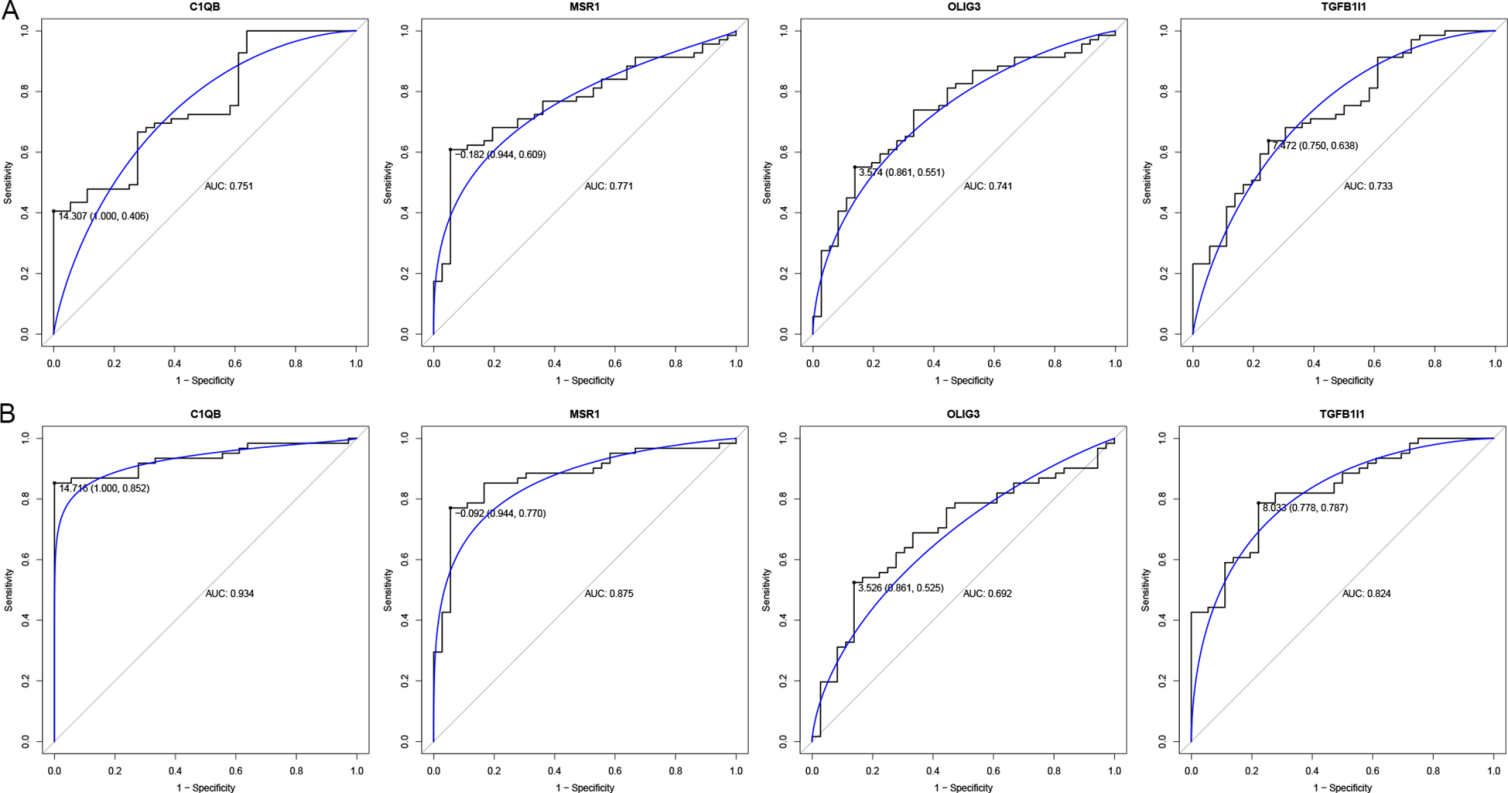
ROC analysis of hub genes in LTBI **(A)** and ATB **(B)**.

The PPI network for common genes was constructed, including 37 nodes and 33 edges (**Figure 2A**). The LASSO regression analysis was performed on 37 genes derived from the PPI network to screen the hub genes. For LTBI, 14 key genes (AQP6, BARHL2, BCAT1, C1QB, CHAC2, CNN1, LHX1, LNX1, MSR1, NPY2R, OLIG3, SOS1, TGFB1I1, and TPX2) were identified (**Figures 2B, C**). For ATB, 7 key genes (C1QB, DDX3Y, LMTK2, MSR1, OLIG3, PDCD1LG2, and TGFB1I1) were identified (**Figures 2D, E**). The key genes in LTBI intersected with those in ATB to yield four hub genes, including C1QB, MSR1, OLIG3 and TGFB1I1 (**Figure 2F**). In LTBI, the AUC values of these hub genes ranged from 0.733 to 0.771, indicating good diagnostic accuracy (**Figure 3A**). In ATB, the AUC values of the hub genes ranged from 0.692 to 0.934, suggesting good diagnostic performance as well (**Figure 3B**).

### 3.3 Characteristics of hub genes in LTBI and ATB

**Figure 4:**
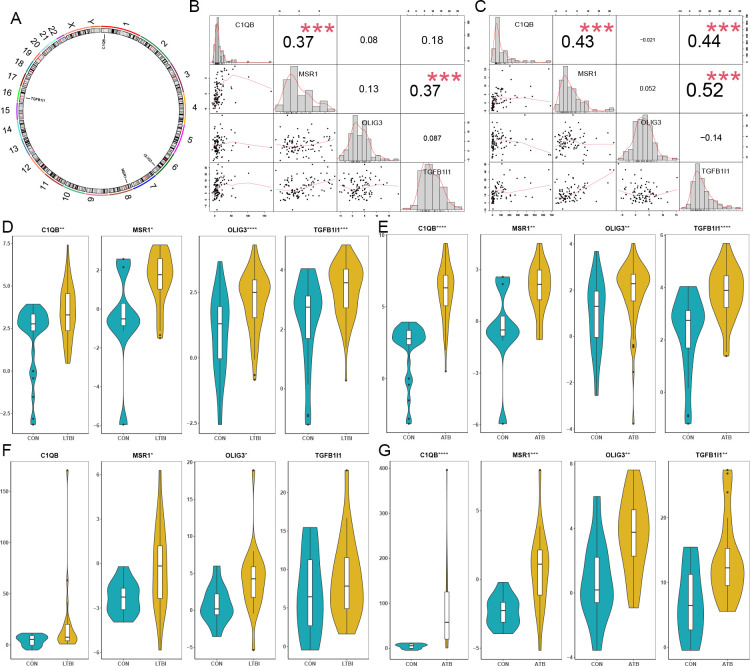
Characteristics of hub genes in LTBI and ATB. **(A)** Distribution of hub genes on the chromosomes. **(B)** Correlation analysis of hub genes in LTBI. **(C)** Correlation analysis of hub genes in ATB. **(D)** The expression levels of hub genes in LTBI in training set. **(E)** The expression levels of hub genes in ATB in training set. **(F)** The expression levels of hub genes in LTBI in validation set. **(G)** The expression levels of hub genes in ATB in validation set. *p <0.05; **p <0.01; ***p <0.001; ****p <0.0001.

The distributions of four hub genes on the chromosomes were exhibited in **Figure 4A**, showing that C1QB, MSR1, OLIG3 and TGFB1I1 are located on chromosome 1, chromosome 8, chromosome 6, and chromosome 16, respectively. Correlation analysis revealed the positive correlations between C1QB and MSR1, MSR1 and TGFB1I1 in both LTBI and ATB, and the positive correlation between C1QB and TGFB1I1 in ATB (**Figures 4B, C**). The box plots exhibited that all four hub genes were significantly up-regulated in LTBI (**Figure 4D**) and ATB (**Figure 4E**) in the training set. In the validation set, four hub genes exhibited similar patterns of increase, with no significance of C1QB and TGFB1I1 in LTBI (**Figures 4F, G**).

### 3.4 Construction of the nomogram

**Figure 5:**
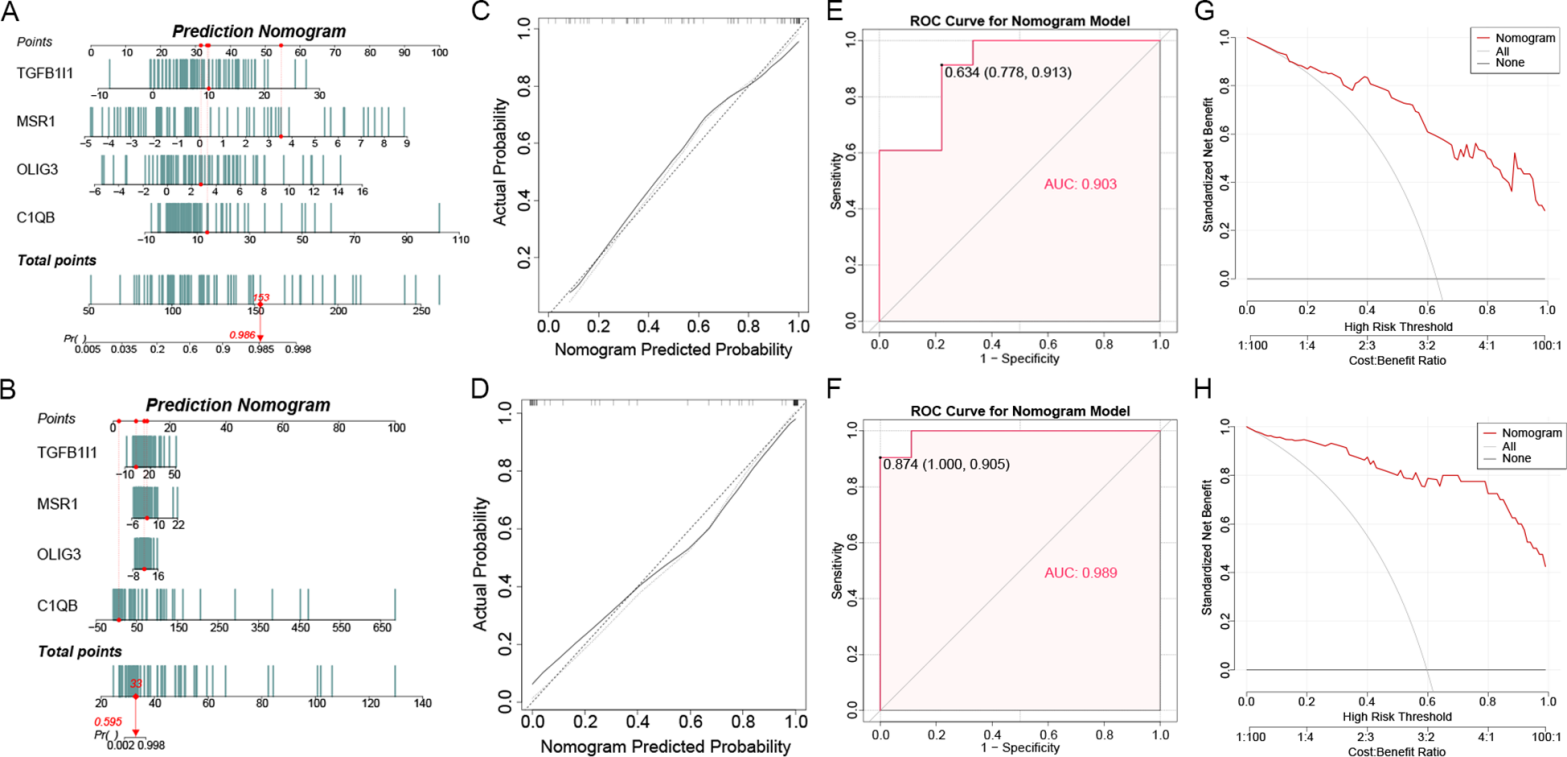
The construction of nomograms for LTBI and ATB. **(A, B)** The nomograms were constructed based on the hub genes for LTBI **(A)** and ATB **(B)**. **(C, D)** The calibration curve of nomograms prediction in LTBI **(C)** and ATB **(D)**. **(E, F)** ROC curve analysis for nomogram models in LTBI **(E)** and ATB **(F)**. **(G, H)** DCA curve for the nomogram models in LTBI **(G)** and ATB **(H)**.

Based on the four hub genes (C1QB, MSR1, OLIG3 and TGFB1I1), the nomogram risk prediction models for LTBI (**Figure 5A**) and ATB (**Figure 5B**) were constructed, respectively. The model integrated the expression profile data of hub genes to achieve quantitative assessment of disease risk. The calibration curve demonstrated the excellent predictive performance of the nomogram models (**Figures 5C, D**). ROC curve analysis indicated that the AUC value of the LTBI nomogram model reached 0.903, and that of the ATB nomogram model was 0.989, demonstrating excellent predictive ability (**Figures 5E, F**). DCA confirmed that nomogram model could provide significant clinical net benefits over a wide range of threshold probabilities, with potential clinical application value (**Figures 5G, H**). These results collectively indicate that the nomogram models based on hub genes could provide a reliable tool for the risk assessment of LTBI and ATB.

### 3.5 GSVA

GSVA results showed that multiple pathways related to immune and inflammatory responses were significantly activated in both LTBI and ATB (**Supplementary Figure 3**), including “TGF_BETA_SIGNALING”, “IL2_STAT5_SIGNALING”, “APOPTOSIS”, “TNFA_SIGNALING_VIA_NFKB”, “INTERFERON_GAMMA_RESPONSE”, “INFLAMMATORY_RESPONSE”, “NOTCH_SIGNALING”, “IL6_JAK_STAT3_SIGNALING”, and “INTERFERON_ALPHA_RESPONSE”. Notably, the hub genes were significantly associated with these pathways (**Supplementary Figure 4**). Specifically, immune and inflammatory-related pathways such as “IL6_JAK_STAT3_SIGNALING”, “INFLAMMATORY_RESPONSE”, “INTERFERON_GAMMA_RESPONSE”, “HALLMARK_NOTCH_SIGNALING”, “TGF_BETA_SIGNALING”, and “TNFA_SIGNALING_VIA_NFKB” were significantly positively correlated with the expression of C1QB, MSR1, OLIG3 and TGFB1I1. These results revealed a highly complex and multi-pathway coordinated immune activation pattern that was shared by LTBI and ATB, characterized by the continuous activation of innate and adaptive immunity, enhanced inflammatory response, and the accompanying immune regulatory mechanisms.

### 3.6 Evaluation of immune characteristics

The infiltration levels of immune cell were evaluated in LTBI and ATB (**Supplementary Figure 5**). The infiltration levels of Activated CD4 T cell, Activated CD8 T cell, Gamma delta T cell, Type 1 T helper cell, Type 17 T helper cell and Type 2 T helper cell were significantly up-regulated in LTBI, while the infiltration level of Macrophage was significantly down-regulated (**Supplementary Figure 5A**), suggesting that the immune system was in a controlled activated state dominated by adaptive immunity (especially T cell response) in the LTBI state. In contrast, in the ATB group, the infiltration levels of Activated B cell, Activated CD8 T cell and Immature B cell were significantly reduced, while Activated dendritic cell, Eosinophil, Gamma delta T cell, Macrophage, Mast cell, MDSC, Monocyte, Natural killer cell, Neutrophil, Regulatory T cell and Type 17 T helper cell showed higher infiltration (**Supplementary Figure 5B**), reflecting the extensive and dysregulated innate immune activation and immunosuppressive microenvironment during the ATB stage. It was noteworthy that Gamma delta T cell and Type 17 T helper cell showed a high infiltration state in both LTBI and ATB, indicating that they may be core participants in the tuberculosis immune response and play stage-specific roles in different disease stages.

**Figure 6:**
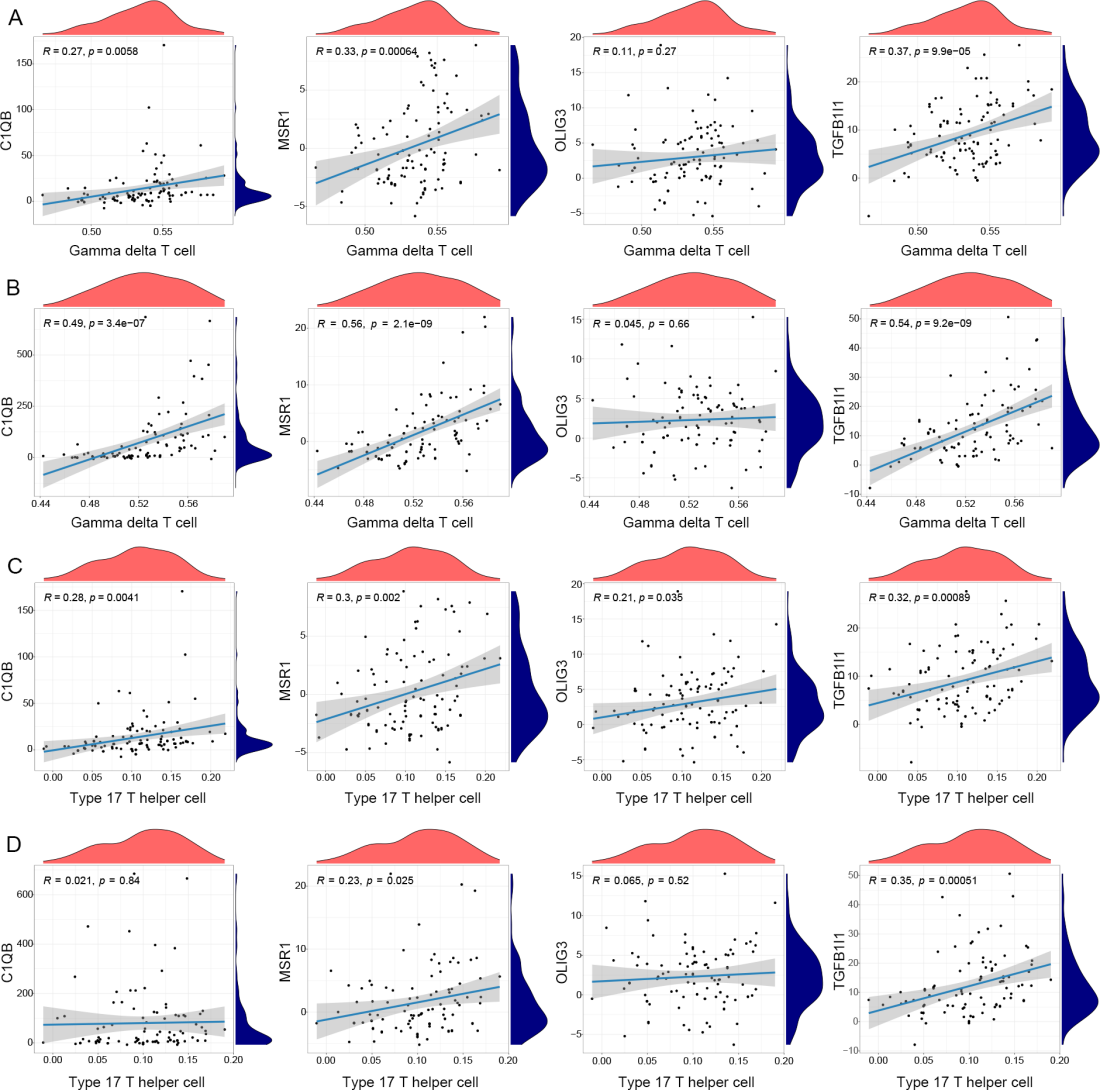
Correlation analysis of immune cells and hub genes. **(A)** Correlation analysis of Gamma delta T cell and hub genes in LTBI. **(B)** Correlation analysis of Gamma delta T cell and hub genes in ATB. **(C)** Correlation analysis of Type 17 T helper cell and hub genes in LTBI. **(D)** Correlation analysis of Type 17 T helper cell and hub genes in ATB.

Further correlation analysis revealed that the infiltration level of Gamma delta T cell was significantly positively correlated with the expressions of C1QB, MSR1, and TGFB1I1 in both LTBI and ATB (**Figures 6A, B**). The infiltration level of Type 17 T helper cell was significantly positively correlated with the expression of C1QB, MSR1, OLIG3 and TGFB1I1 in LTBI, and significantly positively correlated with OLIG3 and TGFB1I1 in ATB (**Figures 6C, D**). The different immune cell infiltration patterns presented by LTBI and ATB may respectively correspond to their specific pathophysiological processes, and these results reveal the potential connection between the immune microenvironment characteristics of LTBI and ATB and the expression of hub genes, laying a solid foundation for further understanding the immune regulatory network in the progression of tuberculosis.

### 3.7 RT-qPCR validation of hub genes

**Figure 7:**
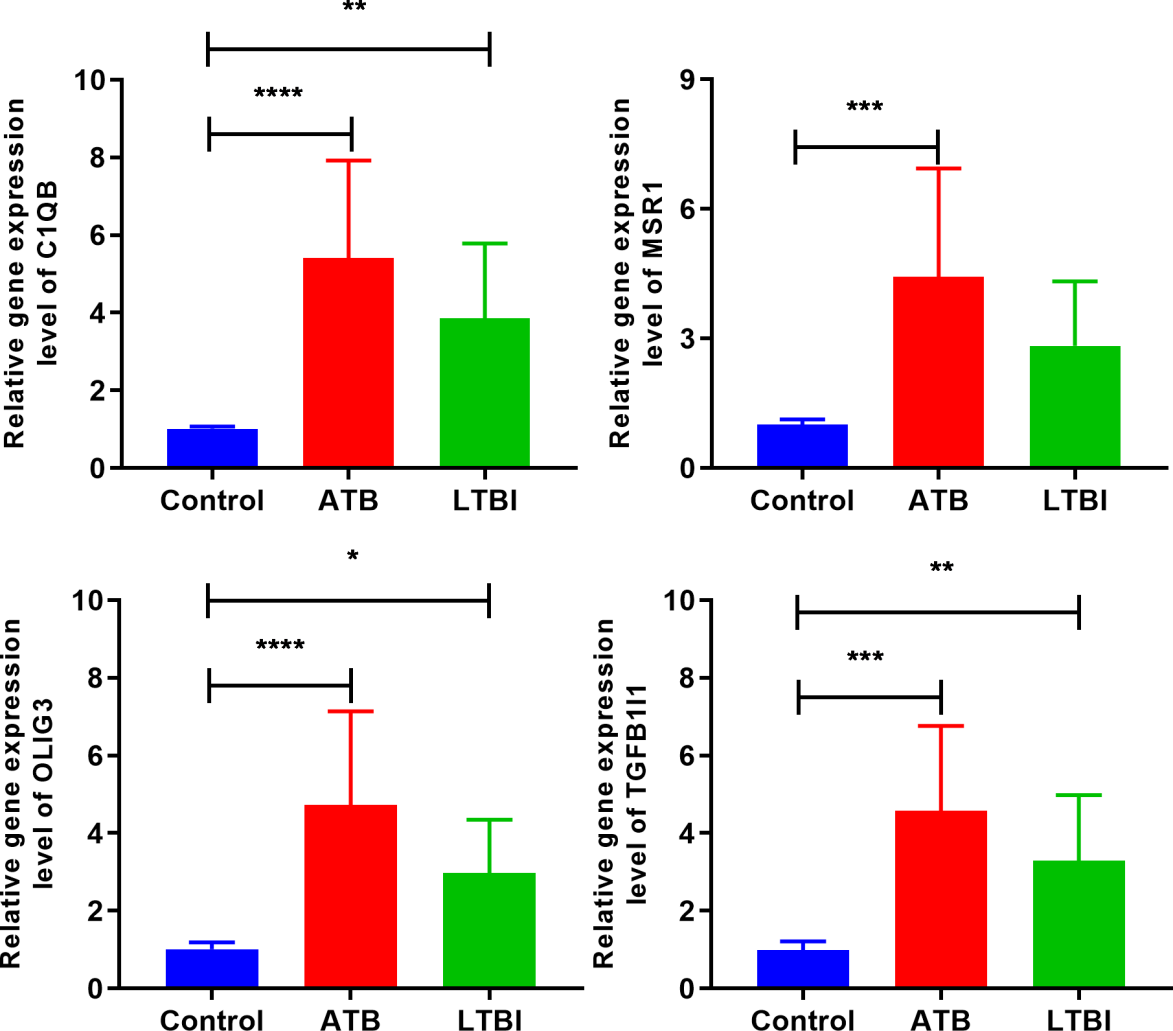
RT-qPCR validation of hub genes. *p <0.05; **p <0.01; ***p <0.001; ****p <0.0001.

The expression levels of four hub genes were validated in human subjects by RT-qPCR (**Figure 7**). Compared with control group, all four hub genes were significantly up-regulated in ATB group, and three hub genes (except MSR1) were significantly up-regulated in LTBI group, which was generally consisted with the integration analysis results.

## 4 Discussion

Infection with *M. tb* triggers a wide range of immune responses within the host, involving complex interactions between the host and the pathogen (Reichmann et al., 2026). In this analysis, functional enrichment analysis and GSVA revealed a highly complex and multi-pathway coordinated immune activation pattern that was shared by LTBI and ATB. Gamma delta T cell and Type 17 T helper cell may be important participants in the tuberculosis immune response. Four hub genes, including C1QB, MSR1, OLIG3 and TGFB1I1, were identified for both LTBI and ATB.

The complement system functions as a critical component of host immune defense that senses danger signals triggered by pathogen- and host tissue damage-associated pattern molecules (Cai et al., 2014). C1QB, encoding a component of the complement 1 (C1q) complex, assembles into the C1q hexamer alongside C1QA and C1QC subunits, a structure that mediates the recognition of immune complexes and initiates the classical complement cascade (Jagatia and Tsolaki, 2021). Elevated C1q expression has been strongly associated with ATB disease and disease severity (Cai et al., 2014). Accumulating evidence highlights the aberrant expression of C1QB across the spectrum of *M. tb* infection. Petrilli et al., reported higher expression of C1QB in ATB than LTBI, which could distinguish ATB from LTBI with high sensitivity and specificity (Petrilli et al., 2020). Using RT-digital PCR, Gliddon et al., further confirmed that C1QB was significantly upregulated in ATB than LTBI (Gliddon et al., 2021). Similarly, Nakiboneka et al., applied RT-qPCR to show that the expression of C1QB was substantially elevated in ATB individuals relative to those with LTBI or healthy controls (Nakiboneka et al., 2024). Wen et al. also showed higher expression of C1QB in ATB than healthy controls via ELISA (Wen et al., 2022). Notably, our study identified C1QB as a shared hub gene for LTBI and ATB, suggesting that C1QB was not only associated with active disease severity but may also play a potential role in maintaining immune homeostasis and regulating disease progression. Elucidating the molecular mechanisms governing C1QB expression could provide valuable insights into how the host immune system balances containment of the pathogen during latency and initiates an effective response during reactivation.

Macrophage is the first cell encountered by mycobacteria within the respiratory tract, serving as the primary host cell and principal effector cell responsible for killing upon activation (Liu and Modlin, 2008). Macrophage scavenger receptor 1 (MSR1), also known as scavenger receptor-A (SR-A), is primarily expressed on the macrophage surface. Given macrophages mediate pathogen elimination through phagocytosis and cytokine production, MSR1 expression is important for the elimination of foreign pathogens (Wang et al., 2021). In spinal cord injury, MSR1-mediated NF-κB signaling pathway facilitates the release of inflammatory mediators (Kong et al., 2020). MSR1 is involved in pathogenic sequestration of cholesterol and foam cell differentiation, a process closely related to the intracellular survival of *M. tb* and chronic infection (Sever-Chroneos et al., 2011). However, Bowdish et al., reported that MSR1 polymorphism has no significant association with susceptibility to tuberculosis (Bowdish et al., 2013). It is reasonable to speculate that MSR1 may exert a dual regulatory role in *M. tb* infection: on one hand, it facilitates the initial phagocytic uptake and pro-inflammatory clearance of *M. tb*; on the other hand, it promotes cholesterol accumulation and foam cell formation that support long-term intracellular mycobacterial persistence. This bidirectional impact may buffer the net effect of MSR1 genetic polymorphisms on tuberculosis susceptibility. Furthermore, genetic redundancy among macrophage scavenger receptors (e.g., MARCO, CD36) likely compensates for loss-of-function MSR1 variants, masking their contribution to innate susceptibility. Therefore, the precise mechanisms by which MSR1 orchestrates phagocytic activity and *M. tb* clearance in macrophages warrant further in-depth investigation.

The oligodendrocyte lineage transcription factor (Olig) family of proteins, consisting of Olig1, Olig2, and Olig3, are basic helix-loop-helix (bHLH) transcription factors expressed in both the developing and mature central nervous system (CNS), which strictly regulate neural cell fate and specification (Szu et al., 2021). At present, direct evidence clarifying the specific function of OLIG3 in tuberculosis is lacking, and whether OLIG3 represents a spurious identification resulting from neural gene enrichment bias in the dataset warrants further investigation. Transforming growth factor beta-1-induced transcript 1 (TGFB1I1), also known as hydrogen peroxide inducible clone 5 (HIC-5), acts as a cofactor of cellular TGF-β1 and promotes focal adhesion formation (Alpha et al., 2020). *In vitro* studies have shown that incubation of human alveolar macrophages with *M. tb* results in increased production of TGF-β (Aung et al., 2005). The spatial atlas of immune responses within human tuberculosis granulomas reveals an IFN-γ-depleted microenvironment enriched for TGF-β, regulatory T cells and IDO1+ PD-L1+ myeloid cells (McCaffrey et al., 2022). TGFB1I1 has been reported to play a key regulatory role in hepatic and pancreatic fibrosis (Lei et al., 2016; Zheng et al., 2023). Formation and healing of mature *M. tb* granulomas involves the differentiation of fibroblasts (Shkurupiy et al., 2014). Based on these findings, we cautiously hypothesize that TGFB1I1 might be involved in the formation and maintenance of granulomas. This provisional hypothesis remains to be verified by further functional experiments and clinical cohort studies before considering its potential value as a therapeutic target for tuberculous fibrosis.

In addition, correlation analysis revealed the positive correlations between C1QB and MSR1, MSR1 and TGFB1I1 in both LTBI and ATB, and the positive correlation between C1QB and TGFB1I1 in ATB. C1QB, a key component of the classical complement pathway, is predominantly expressed in myeloid immune cells (e.g., macrophages, dendritic cells) and mediates phagocytosis and inflammatory regulation (Jagatia and Tsolaki, 2021). MSR1 encodes macrophage scavenger receptor 1, which directly mediates the phagocytosis of pathogens (including *M. tb*) and pro-inflammatory signaling (Petrilli et al., 2020). Their strong positive correlation suggests that they may jointly participate in the macrophage-mediated innate immune response in the context of tuberculosis infection. TGFB1I1 is a downstream effector molecule of the TGF-β signaling pathway, involved in cell adhesion, migration and immune regulation (Xu et al., 2024). The positive correlation between C1QB/MSR1 and TGFB1I1 may suggest that the complement-phagocytosis pathway interacts with the TGF-β-mediated immune regulatory network, and plays a coordinating role in chronic inflammation or immune homeostasis maintenance during tuberculosis infection. Although the RT-qPCR validation supports the reliability of most hub genes identified in our bioinformatic analysis, a discrepancy was observed for MSR1, which may be attributed to differences in detection platforms, the inherent immunological heterogeneity of LTBI individuals, and variations in peripheral blood immune cell subset composition between cohorts. In addition, the small sample size of the validation cohort represents a major limitation of this study, which may reduce the statistical power and generalizability of our validation results. It is necessary to conduct larger-scale cohort studies and functional experiments in the future to further confirm the expression patterns and mechanisms of these hub genes in LTBI and ATB.

In conclusion, our study identified four hub genes for LTBI and ATB and revealed complex regulatory mechanisms in tuberculosis immune response. This study may provide a novel perspective for understanding the pathological characteristics of tuberculosis and opens up new directions for the diagnosis and treatment of tuberculosis. Further experiments are advocated to validate the hub genes in tuberculosis.

## Data Availability

The original contributions presented in the study are included in the article/**Supplementary Material**. Further inquiries can be directed to the corresponding author.
