# Supplementary material for: Exploration of common immune mechanisms and hub genes in latent and active tuberculosis infection
Source: Front Cell Infect Microbiol. 2026 May 20;16:1798990. doi: 10.3389/fcimb.2026.1798990 (PMC13231278; doi:10.3389/fcimb.2026.1798990)
Supplement: Supplementary file 1 [file Table1.docx]

***Supplementary Materials***

**Supplementary Figures**


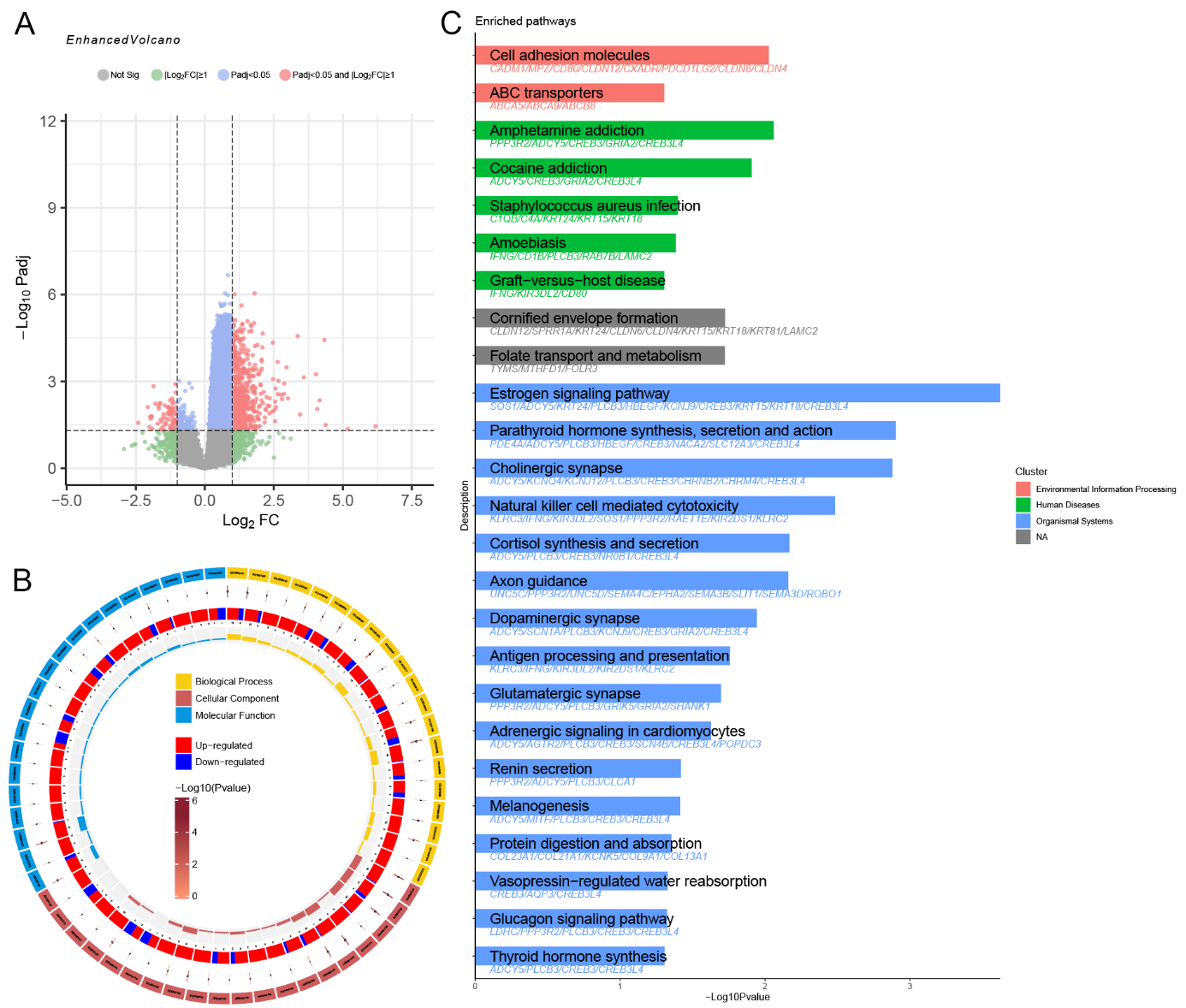


**Figure S1** Identification and functional enrichment analysis of DEGs in LTBI.

(A) Volcano map of DEGs in LTBI. (B) GO analysis of DEGs in LTBI. (C) KEGG analysis of DEGs in LTBI.


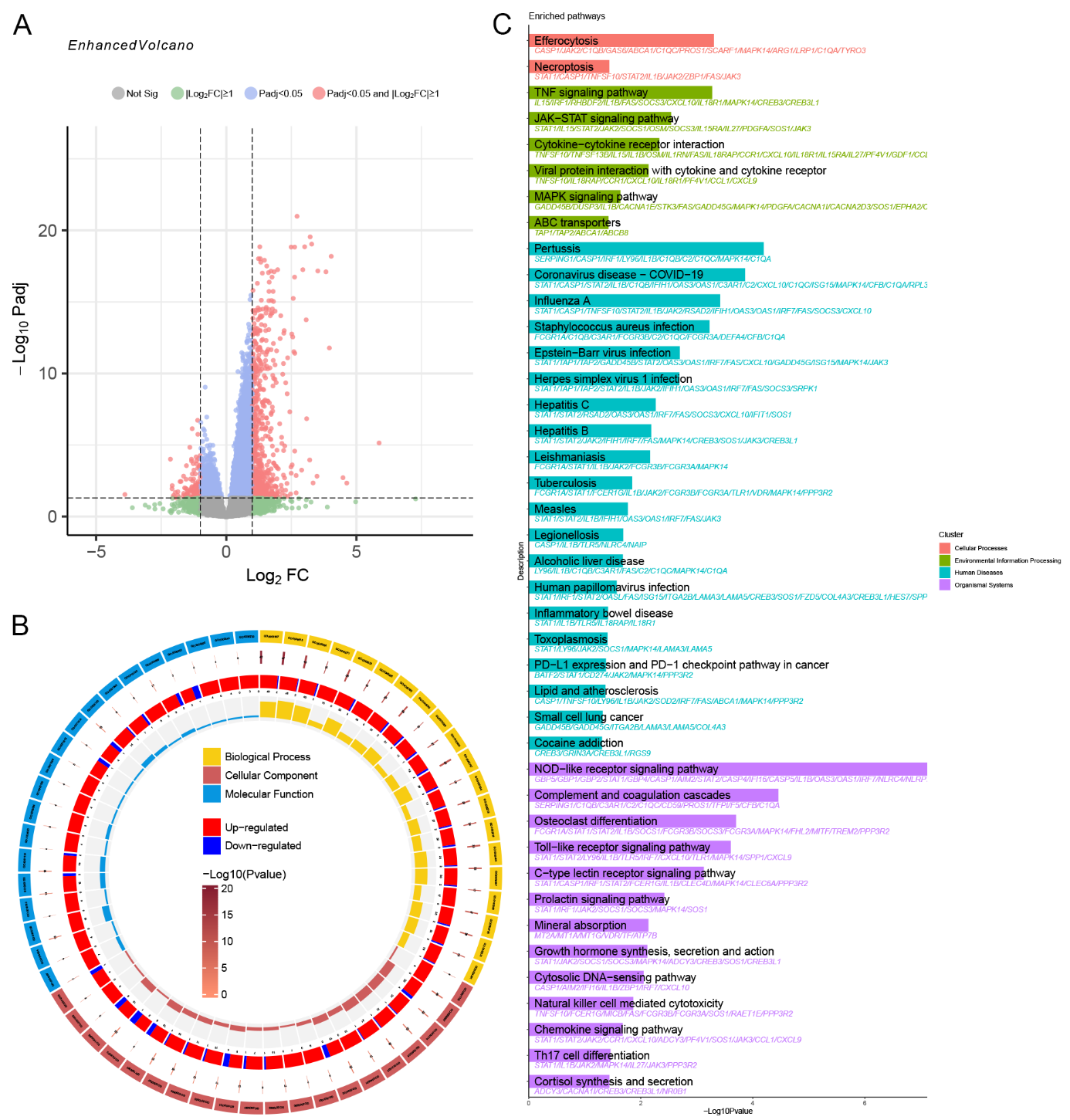


**Figure S2** Identification and functional enrichment analysis of DEGs in ATB.

(A) Volcano map of DEGs in ATB. (B) GO analysis of DEGs in ATB. (C) KEGG analysis of DEGs in ATB.


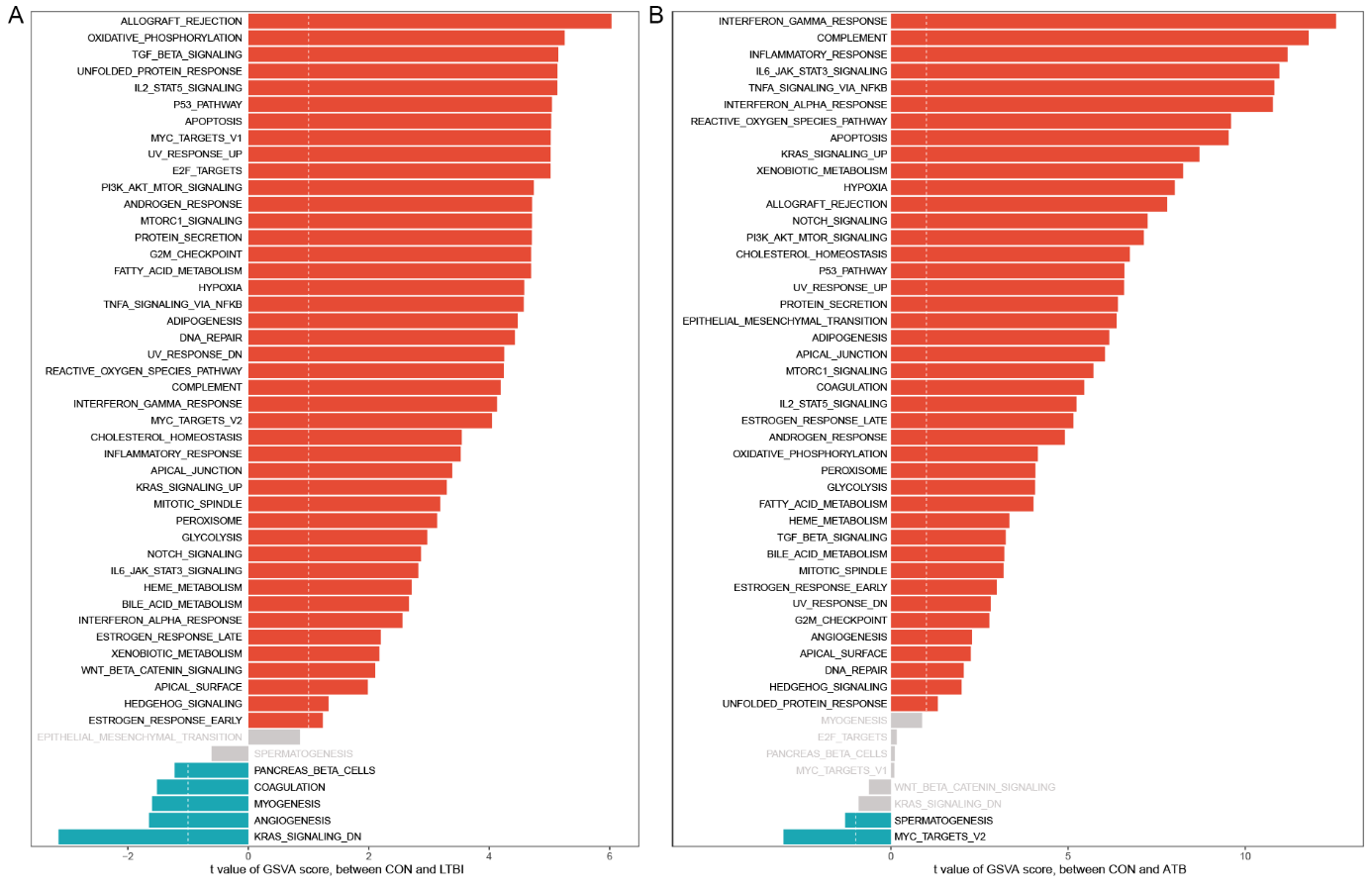


**Figure S3** Gene set variation analysis in LTBI (A) and ATB (B).


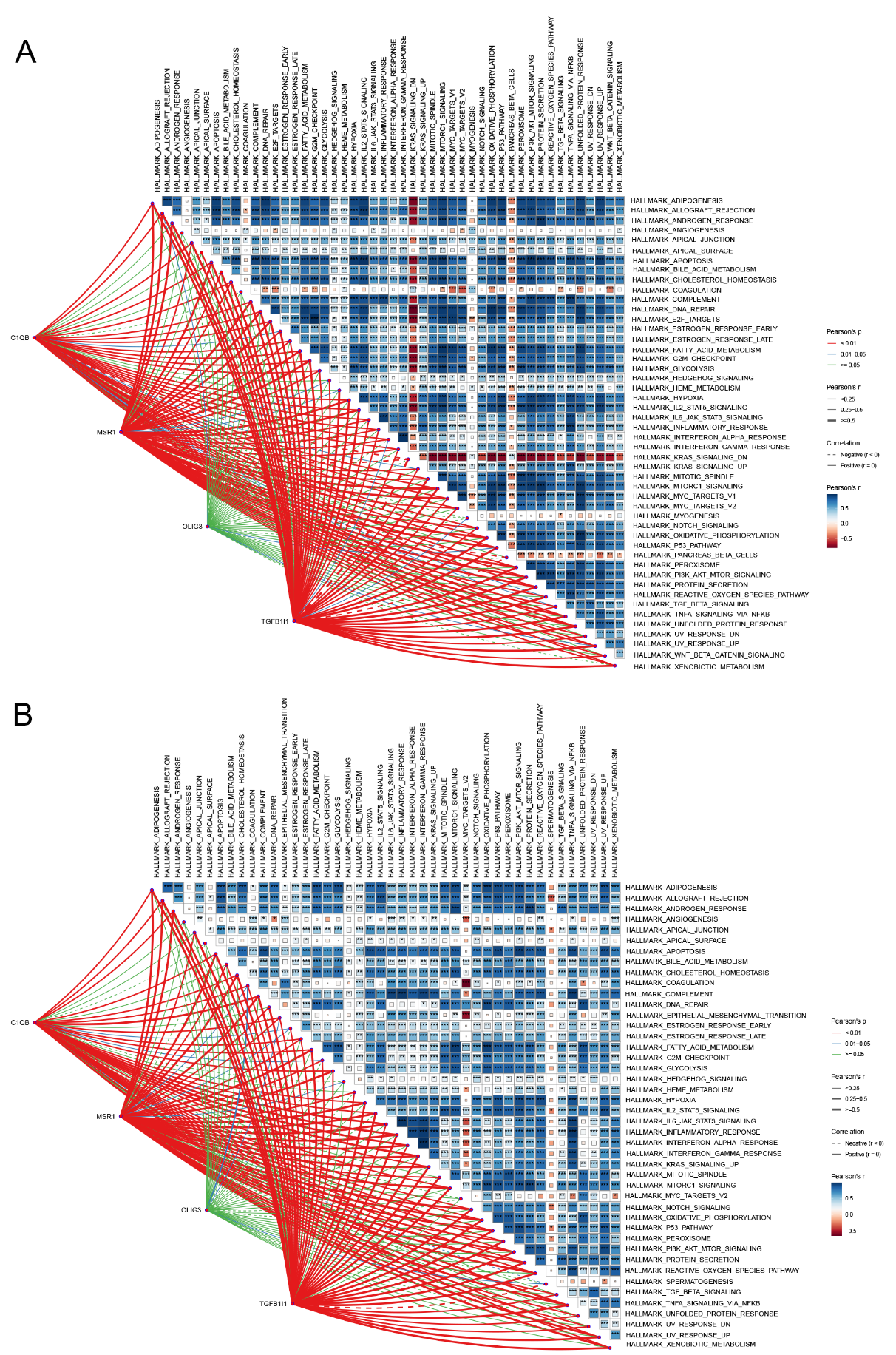


**Figure S4** The correlation analysis between hub genes and the pathways activated in LTBI (A) and ATB (B).


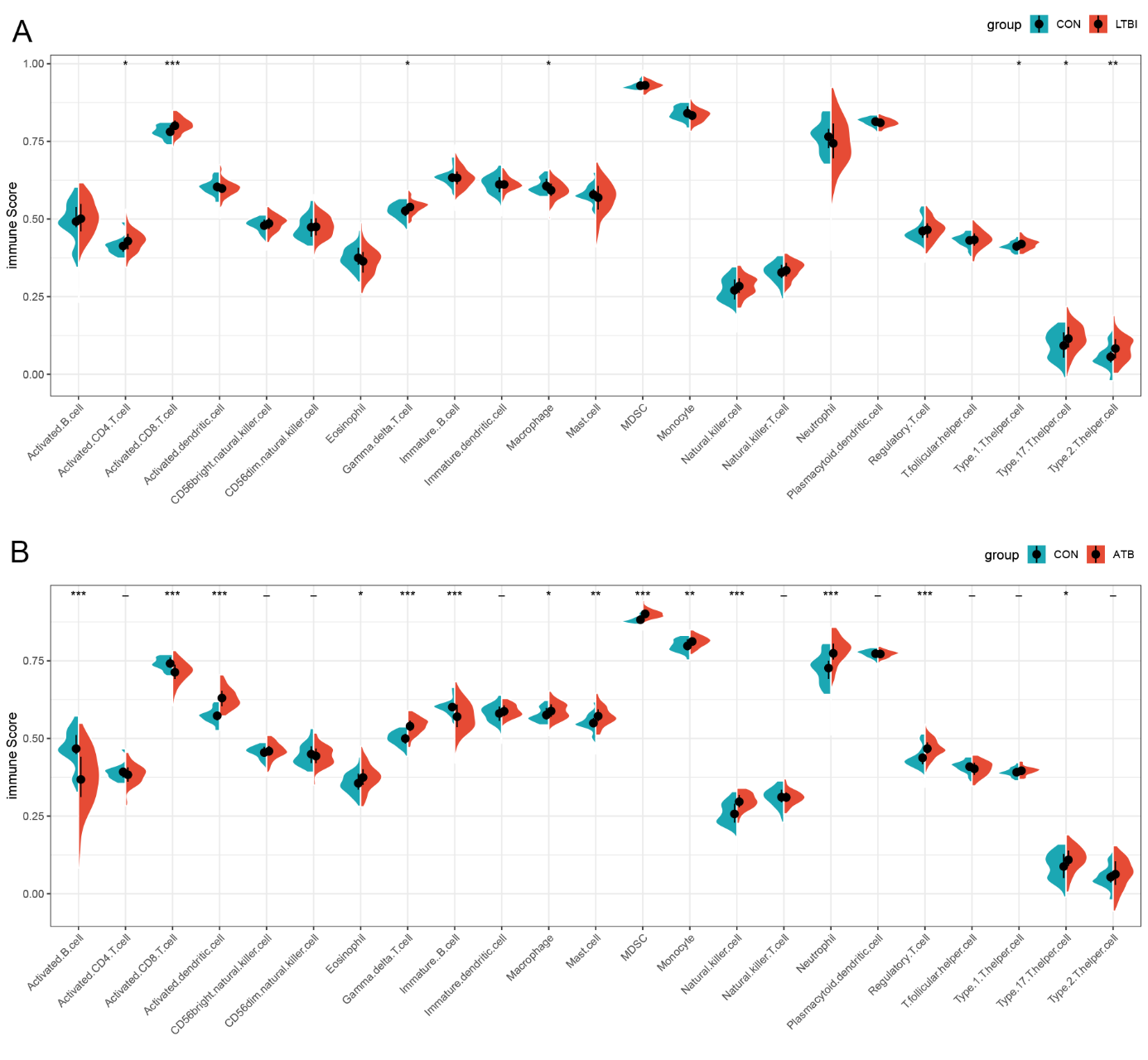


**Figure S5** Immune characteristics in LTBI (A) and ATB (B).
