# Supplementary material for: Exploration of common immune mechanisms and hub genes in latent and active tuberculosis infection
Source: Front Cell Infect Microbiol. 2026 May 20;16:1798990. doi: 10.3389/fcimb.2026.1798990 (PMC13231278; doi:10.3389/fcimb.2026.1798990)
Supplement: Supplementary file 2 [file Table2.docx]

**Supplementary Table**

**Table S1** The characteristics of individuals in GEO datasets used in this study

|  | Overall | Control | LTBI | ATB | p-value |
| --- | --- | --- | --- | --- | --- |
| GSE19491 | n = 166 | n = 36 | n = 69 | n = 61 |  |
| Age | 32.17 (12.46) | 30.53 (7.81) | 29.00 (11.17) | 36.72 (14.68) | 0.001 |
| Gender |  |  |  |  | 0.017 |
| Female | 84 (51%) | 22 (61%) | 40 (58%) | 22 (36%) |  |
| Male | 82 (49%) | 14 (39%) | 29 (42%) | 39 (64%) |  |
| Ethnicity |  |  |  |  | <0.001 |
| Asian other | 18 (11%) | 4 (11%) | 10 (14%) | 4 (6.6%) |  |
| Black | 82 (49%) | 6 (17%) | 45 (65%) | 31 (51%) |  |
| Other | 6 (3.6%) | 2 (5.6%) | 2 (2.9%) | 2 (3.3%) |  |
| South Asian | 24 (14%) | 6 (17%) | 7 (10%) | 11 (18%) |  |
| White | 36 (22%) | 18 (50%) | 5 (7.2%) | 13 (21%) |  |
| GSE19444 | n = 54 | n = 12 | n = 21 | n = 21 |  |
| Age | 37.50 (15.26) | 31.00 (9.07) | 36.29 (13.45) | 42.43 (18.41) | 0.2 |
| Gender |  |  |  |  | 0.5 |
| Female | 26 (48%) | 7 (58%) | 11 (52%) | 8 (38%) |  |
| Male | 28 (52%) | 5 (42%) | 10 (48%) | 13 (62%) |  |
| Ethnicity |  |  |  |  | 0.6 |
| Asian other | 8 (15%) | 2 (17%) | 5 (24%) | 1 (4.8%) |  |
| Black | 15 (28%) | 3 (25%) | 7 (33%) | 5 (24%) |  |
| Other | 4 (7.4%) | 1 (8.3%) | 2 (9.5%) | 1 (4.8%) |  |
| South Asian | 13 (24%) | 3 (25%) | 4 (19%) | 6 (29%) |  |
| White | 14 (26%) | 3 (25%) | 3 (14%) | 8 (38%) |  |

LTBI, latent tuberculosis infection; ATB, active tuberculosis.
